# Sudden Sensorineural Hearing Loss Associated with Oral Consumption of Hand Sanitizer: A Case Report 

**DOI:** 10.22038/IJORL.2023.70374.3390

**Published:** 2023-05

**Authors:** Arshit Kataria, Siddharth Jain, Manika Arora, Sonal Mishra

**Affiliations:** 1 *Department of ENT and * *Head & Neck Surgery* *, All India Institute of Medical Sciences, Bathinda, Punjab, India.*; 2 *Department of Orthopaedics, All India Institute of Medical Sciences, Bhopal, Madhya Pradesh, India.*; 3 *Department of Head and Neck Surgery, Government Medical College, Patiala,* *Punjab, India.*; 4 *Department of Anaesthesiology, S.M.S. Medical College, Jaipur, Rajasthan, India.*

**Keywords:** Alcohol, Case report, Coronavirus, Sudden sensorineural hearing loss (SSNHL), Sanitizer

## Abstract

**Introduction:**

Pediatric sudden sensorineural hearing loss (SSNHL) is a rare otological emergency. With the emergence of the Coronavirus 19 pandemic, alcohol-based hand sanitizers are among the essential household items. Many hand sanitizers are frequently coupled with scents that young children may find pleasant.

**Case Report:**

A 5-year-old girl presented to our clinic with hearing loss after the consumption of alcohol-based hand sanitizer. A pure tone audiogram showed bilateral SSNHL. The child was prescribed systemic corticosteroids resulted in a slight improvement in hearing thresholds. The child was followed up at 6 and 18 months showing no further improvement in hearing thresholds.

**Conclusion:**

Although various infective, vascular, and immune responses have been proposed, alcohol-based hand sanitizer consumption has not been reported to present with SSNHL to the best of our knowledge. In the current scenario of the Coronavirus pandemic, otorhinolaryngologists must keep in mind that SSNHL may occur as a result of hazardous alcohol-based hand disinfectant consumption.

## Introduction

Sudden sensorineural hearing loss (SSNHL) is most commonly defined as a sensorineural hearing loss of 30dB or greater over at least three contiguous audiometric frequencies occurring within a 72-hr period (1). Sometimes SSNHL is accompanied by tinnitus or vertigo. The presentation of SSNHL in the pediatric group is uncommon and requires a careful review of history (2).

The COVID-19 (Coronavirus Disease-2019) pandemic has become a major worldwide public health concern, prompting widespread usage of hand disinfectants due to its contagious nature. Hand sanitizers are typically divided into two types: alcohol-based (ABHS) and alcohol-free. 

An ABHS may contain up to 60%-95% ethanol or isopropyl alcohol by volume, to be applied to the hands to eliminate microorganisms and temporarily limit their multiplication. ABHS can efficiently and swiftly eliminate germs over a broad antimicrobial spectrum without the need for water or towel drying. Alcohol-free sanitizers consist of specialized chemicals having antimicrobial activity (3). 

Many hand sanitizers are frequently coupled with scents that young children may find pleasant. Alcohol hand sanitizer consumption is linked to worse consequences than nonalcohol hand sanitizer exposure (4).

Alcohol is quickly absorbed into the circulation from the stomach and small intestine (5). It has been associated with hearing loss by causing disturbances in cochlear blood flow, particularly hypoperfusion and possible ischemia (6). 

The cytotoxic effect of ethanol on vestibulocochlear ganglion causing hearing and balancing problems have been established (7). There have been no instances of abrupt hearing loss caused by sanitizer ingestion in either adult or pediatric patients, to the best of our knowledge. We have described the case of a 5-year-old child who acquired bilateral SSNHL after the consumption of sanitizer. This case report was prepared following the CARE guidelines (8).

## Case Report

A 5-year-old female patient presented to pediatric emergency in June 2021 with chief complaints of nausea and vomiting for the last 6 hours with a history of consumption of sanitizer orally. According to the history given by her parents, she was drinking liquid from a bottle that was being used for sanitization during the ongoing Coronavirus pandemic. It contained 150 ml of liquid sanitizer consisting of Ethyl alcohol I.P 70% v/v with emollients and moisturizer. She had consumed 50-60 ml according to her parents. 

The general condition of the patient was fair. The patient was admitted to the pediatrics department and started intravenous fluids. All routine investigations (hemogram, liver function tests, renal function tests, C reactive protein) were sent to our laboratory and were at normal levels. The patient underwent gastric lavage. The ENT department was consulted for hearing loss developed while discharging the patient after 24 hours.

A detailed history was taken, there was no history of hard of hearing before. Neonatal records of the patient were reviewed showing normal neonatal screening tests. Her speech and language milestones were normal. There was no history of tinnitus or vertigo. 

Also, there was no history of any medications other than intravenous fluids during admission to the pediatrics department before hearing loss. There was no past history pertaining to any neurological disorder or developmental abnormality. Otoscopy and other ENT examinations were normal. 

**Fig 1:**
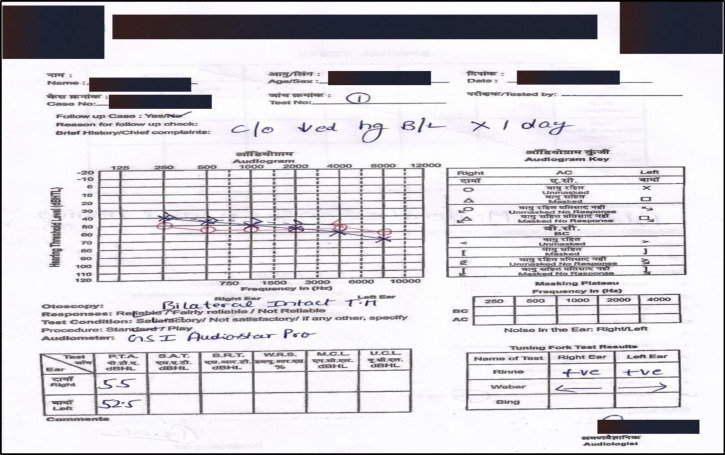
Pure tone audiometry findings of the patient at the initial presentation

The patient underwent pure tone audiometry showing a bilateral sudden sensorineural hearing loss. Pure tone averages of 0.5, 1, 2, 4 kHz (9) for the right and left ears were 55 dB and 52 dB respectively (Figure 1). 

Speech Discrimination Score (SDS) was 75% and 78% in the right ear and left ear respectively. Speech Reception Threshold (SRT) was 55 dB and 50 dB in the right ear and left ear respectively. Tympanometry showed Type A curve. 

**Fig 2:**
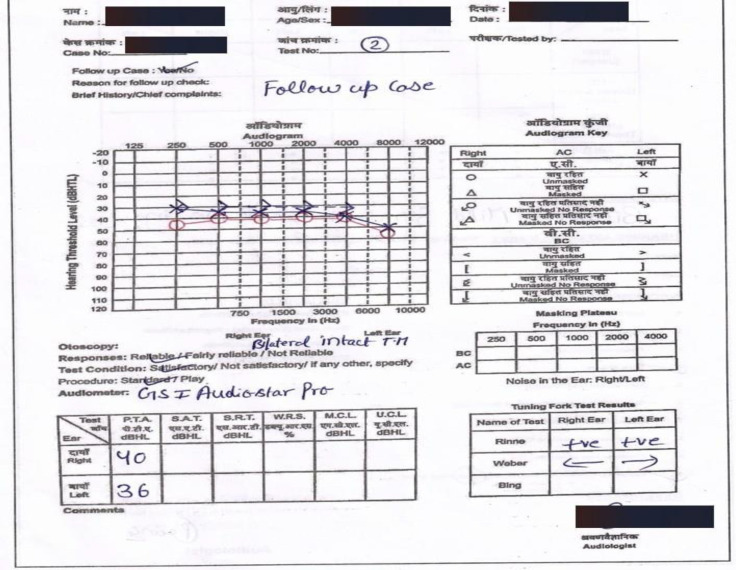
Pure tone audiometry findings of the patient after steroid therapy (11^th^ day)

The patient was prescribed oral prednisolone of 1mg/kg/day for 10 days and then tapered. The patient was followed up with alternate-day audiogram, hearing improvement was around 15 dB comparing 1^st^ and 11^th^ day audiograms. On the 11^th^ day, Pure tone averages of 0.5, 1, 2, 4 kHz for the right and left ears were 40 dB and 36 dB (Figure 2). 

SDS was 85% & 90% and SRT was 40 dB & 35dB in the right and left ear respectively on the 11^th^ day. The patient was prescribed bilateral conventional hearing aids. The patient was followed up after 6 months and 18 months, audiometric results were approximately the same as on 11^th^ day.

However, the major limitation of this case report is that we couldn’t use high frequency for a hearing evaluation. Also, we didn’t evaluate the disability by using any questionnaire.

## Discussion

Studies on SSNHL in the pediatric population are sparse and have poor treatment outcomes (10). Common causes attributed to SSNHL are viruses (mumps, herpes, cytomegalovirus), vascular causes, autoimmune diseases, endolymphatic hydrops, trauma, and ototoxicity (11). 

Pitaro J et al in a study established viruses as the most common etiology for SSNHL in the pediatric group (12). On reviewing various works of literature, we have found some unusual etiological factors associated with SSNHL (Table 1)(13-15). 

Demir E et al. reported a case of a 6-year-old girl developing bilateral SNHL after drinking electronic cigarette liquid. 

The patient was treated with intravenous methylprednisolone 1mg/kg in tapering dose for 10 days with daily audiogram follow-up, with approximately 20 dB of hearing improvement (16). The nicotine contained in an electronic cigarette was thought to be responsible for sensorineural hearing loss. In our case, the cytotoxic effects of ethanol are thought to be the cause of sudden sensorineural hearing loss (6,7).Stachler RJ et al. in a study described the recommended dosage of Prednisolone as 1mg/kg/day for 10-14 days. (17). In our case treatment with oral prednisolone (1mg/kg/day) for 10 days showed approximately 15 dB of hearing improvement. In a study by Kim JY et al, pediatric SSNHL patients were given a high dose of systemic steroids, and the recovery rate was 55.2% and concluded high threshold hearing loss at initial presentation was a poor prognostic factor (10). Initial severe hearing loss, associated vertigo, and a ‘downward’ audiometric curve are poor prognostic factors as described by Roman S et al.(18). Children with SSNHL are managed by audiological investigations and magnetic resonance imaging as in adults. Systemic or intratympanic steroids and hyperbaric oxygen therapy are the mainstays in the pediatric population (19). 

**Table 1:** Summarization of the association of some unusual etiologies with Sudden Sensorineural hearing loss

**Article**	**Year of publication**	**Unusual etiology**	**Treatment**	**Hearing outcomes**
Demir E et al.(16)	2018	Nicotine in electronic cigarettes	Intravenous corticosteroids	SSNHL with 63 dB and 67 dB of pure tone averages (right and left respectively), approximately 20 dB of hearing improvement following treatment
Kang SM et al.(13)	2020	Hemodialysis in renal failure (osmotic alterations)	Oral steroid therapy and/or intratympanic steroid injection.	Bilateral SSNHL, 36.4% patients achieved either a complete or partial recovery following treatment
Jrad M et al.(14)	2021	Intracochlear hemorrhage	Oral steroids Peripheral vasodilator Antivirals Hyperbaric oxygen therapy	SSNHL in left ear, no improvement following treatment
Mughal A et al.(15)	2022	Dengue fever	Oral steroid therapy	SSNHL with pure tone averages of 90dB, improved to 60dB in left ear following treatment
Current study	2023	Ethanol in sanitizers	Oral steroid therapy	SSNHL with 55 dB and 52 dB of pure tone averages (right and left respectively), approximately 15 dB of hearing improvement following treatment

Accidental ingestion of hand disinfectants causes an increase in blood alcohol levels leading to apnea, acidosis, delirium, vomiting, abdominal pain, nausea, throat irritation, drowsiness, loss of consciousness, and even death (20-22). 

In 2013, the Victorian Poisons Information Centre received 15729 calls pertaining to children under the age of five, with topical antiseptics/hand sanitizers being the seventh most common source of poison to which this age group was exposed (23). 

However, as in our case, unanticipated side effects can occur. Using alcohol hand sanitizers carefully, under adult supervision, and with suitable child safety precautions, as well as keeping them out of the reach of small children, may help to avoid unforeseen negative repercussions.

## Conclusion

Sudden sensorineural hearing loss is a medical emergency. Prompt diagnosis and early intervention may prevent permanent hearing loss. In the current scenario of coronavirus pandemic, otolaryngologists must keep in mind that SSNHL may occur as a result of hazardous alcohol-based hand disinfectant consumption.

*Patient’s parent’s perspective*

Our child consumed some amount of sanitizer after which she had nausea and vomiting for which we visited the hospital. After receiving treatment, she was well, but the next day she was not able to hear our words properly for which we were referred to ENT specialist. 

We were asked various questions regarding the birth and growth of the child and our child underwent various investigations. We got worried after knowing that investigations are in favor of sensorineural hearing loss. After consulting the ENT specialist, our child was given oral drug therapy and had hearing tests conducted every alternate day. After the completion of treatment, our child had a slight improvement in hearing. 

The doctor advised us to use hearing aids for both ears for proper speech and language development of the child and we were advised to follow up regularly for a hearing evaluation. We are thankful to doctors for guiding us.
